# Preventing RSV Infection in Children: Current Passive Immunizations and Vaccine Development

**DOI:** 10.3390/pathogens14020104

**Published:** 2025-01-21

**Authors:** Pius I. Babawale, Iván Martínez-Espinoza, Alaine’ M. Mitchell, Antonieta Guerrero-Plata

**Affiliations:** Department of Pathobiological Sciences, Louisiana State University, Baton Rouge, LA 70803, USA; pbabaw1@lsu.edu (P.I.B.); imart27@lsu.edu (I.M.-E.); amitc91@lsu.edu (A.M.M.)

**Keywords:** respiratory syncytial virus, RSV, vaccine, RSV pediatric vaccine, RSV prophylaxis

## Abstract

Human respiratory syncytial virus (RSV) is a leading cause of acute respiratory tract infection and lower respiratory tract infection, associated with high morbidity and mortality in young children, the elderly, and immunocompromised individuals. Initial attempts to develop an RSV vaccine in the 1960s were faced with a setback due to the enhanced RSV disease developed by vaccinated children. More recent advancements have led to the generation of RSV vaccines for older adults and pregnant women. However, there are still no commercially available RSV vaccines for infants. This work summarizes the current passive immunizations and the ongoing efforts to develop an RSV vaccine for infants.

## 1. Introduction

Human respiratory syncytial virus (RSV, HRSV) is a non-segmented, negative-sense single-stranded RNA virus that belongs to the *Pneumoviridae* family [1,2], causing respiratory illnesses globally, mainly in children, adults, and immunocompromised individuals. By the age of two, it is estimated that nearly all children have had at least one episode of RSV [3]. RSV symptoms range from mild upper respiratory tract infections to severe lower respiratory tract infections like bronchiolitis and pneumonia. RSV is responsible for approximately 48.9% of acute respiratory infections in children under one year old [4]. There are an estimated 3.6 million hospitalizations and over 100,000 deaths annually as a result of RSV-related lower respiratory tract infections in children under five years of age worldwide [5,6]. Various factors influence disease severity, including age, underlying health conditions, and prematurity. In pediatric patients, severe RSV illness is frequently linked to prematurity, bronchopulmonary dysplasia (BPD), or congenital heart disease [7]. In children, it causes bronchiolitis, often necessitates intensive care, and even leads to death. Additionally, RSV can trigger pneumonia in the elderly. Severe bronchiolitis, primarily caused by RSV, has led to increased healthcare utilization, including oxygen therapy and more extended hospital stays. RSV is a trigger for airway inflammation, and it is associated with wheezing and bronchial hyperreactivity [8]. Studies have also shown that children who experience a severe RSV infection in their first year of life have a higher likelihood of developing asthma later on [9]. RSV significantly impacts older and high-risk adults, causing 4.66–7.80% of symptomatic respiratory infections with notable case fatality proportions, emphasizing the need for increased monitoring and research for improved management [10].

RSV was first identified in 1956, and due to its significant impact on infants, the elderly, and immunocompromised individuals, efforts to develop a vaccine followed shortly and have been ongoing for decades. Early attempts to generate an RSV vaccine for children in 1967 using a formalin-inactivated RSV vaccine were met with tragic outcomes, as vaccinated children experienced enhanced respiratory disease upon subsequent natural RSV infection, leading to hospitalizations and fatalities [11]. These events created a significant setback and delayed further vaccine development efforts. As of 2024, 55 years after the first attempt to develop an RSV vaccine, there are three U.S. Food and Drug Administration (FDA)-approved RSV vaccines for the elderly and pregnant women. In contrast, there are no active immunizations available for children, one of the most at-risk populations for RSV infection. The pediatric population remains highly vulnerable, as observed recently in the fall of 2022, when a surge in RSV-related illness increased hospitalization in infants [12]. Although passive immunizations are used in infants, such as maternal immunization and monoclonal antibodies against RSV, a pediatric vaccine is needed. Over the years, RSV vaccine design has shifted to structure-based design, and newer technologies leverage a deeper understanding of the structure of RSV proteins, together with immune response mechanisms.

RSV has a 15.2 kb genome comprising 10 genes that encode 11 proteins [13,14,15]. It has three proteins expressed on the virus surface (G, F, and SH), three proteins that are part of the viral nucleocapsid (P, N, and L), three matrix proteins (M, M2-1, M2-2), and two non-structural proteins (NS1, NS2) that are only expressed in the infected cells [16,17] RSV uses the G protein for the initial attachment to the ciliated respiratory epithelial cell, the primary target for viral replication, and the F protein for fusion to the target cell membrane [13].

Upon RSV natural infection, the infected host initially mounts an innate immune response by activating innate cells such as macrophages that help in phagocytosis and dendritic cells, antigen-presenting cells crucial for T cell priming and differentiation [18]. After a few days, the adaptive immune response (both humoral and cellular) is activated. The humoral response produces RSV-specific neutralizing antibodies, primarily targeting the RSV F and G proteins. The G and F proteins are responsible for the initial attachment and fusion of the virus with host cells, respectively. Moreover, the F protein is conserved across RSV strains, making it the primary target for neutralizing antibodies and an attractive target for vaccine development [19,20]. However, the antibody titers often wane within months, leading to incomplete protection and the possibility of reinfection within the same season. The cellular immune response involving T cells plays a crucial role in viral clearance. Cytotoxic T lymphocytes (CD8^+^ T cells) target infected cells to eliminate the infection, while helper T cells (CD4^+^ T-cells) produce cytokines that enhance macrophage and dendritic cell activity [21]. However, despite the induction of the immune response, it can be insufficient to prevent reinfection, and in some cases, it can contribute to immunopathology, exacerbating disease severity. Although vaccination aims to elicit a protective immune response against a pathogen without causing disease, some challenges remain in the case of RSV vaccine development. Historically, RSV vaccine formulation has led to enhanced RSV disease (ERD) upon subsequent natural infection in young children [11], raising a vaccine safety concern. Therefore, most RSV vaccines in clinical trials are now designed to induce a robust RSV-specific neutralizing antibody response while minimizing immunopathology.

Multiple approaches, including live-attenuated [22], subunit [23], vector-based [24], and mRNA [25] RSV vaccines, have now been explored, each presenting unique challenges and potential benefits. Currently, there are multiple RSV vaccine candidates for children in different stages of development. Therefore, this review aims to bring together the earlier challenges faced in RSV pediatric vaccine development, the current passive immunizations available, and the ongoing efforts to develop active RSV vaccination for infants.

## 2. Early Attempts and Setbacks in the Development of Pediatric RSV Vaccine

The development of RSV vaccines has faced significant challenges, mainly due to early setbacks in the 1960s. Because of the success acquired by the inactivated polio vaccine that was approved in 1955, researchers in the U.S. developed a formalin-inactivated RSV vaccine, and four large studies were conducted between 1965 and 1966. The participating infants were immunized before 6 months of age and before RSV exposure. Out of the total 31 infants vaccinated, 20 got infected with natural RSV infection, and out of these RSV-infected infants, 16 were hospitalized and two died [26]. The major severe adverse effects encountered with the formalin-inactivated RSV vaccine were ERD and fatalities among previously unexposed vaccinated infants [27]. ERD occurs when the symptoms worsen after a natural RSV infection due to prior vaccination against RSV, characterized by a Th2-biased immune response, which is a hallmark of enhanced RSV disease and eventually leads to allergic airway hyperresponsiveness and increased eosinophilia [28]. The characterization of the clinical presentation of the two vaccinated infants that died as a result of ERD showed prolonged cough, tachypnea, rhinorrhea complicated by high fever, and bronchiolar infiltration predominantly with eosinophils and neutrophils [27]. Multiple mechanisms have been suggested to contribute to the adverse effects of the RSV vaccine and ERD, including the effect of the formalin on the RSV antigens [29] and poor affinity maturation [30]. The observed adverse effects led to a significant setback and halted further development for decades [31]. Subsequent vaccine efforts were largely hindered as the scientific community grappled with the implications of vaccine-enhanced disease [31]. Other challenges faced during the early days of RSV vaccine development include the biology of the virus, short time to first exposure, maternal antibodies interference, and Th2 immune bias in infants, hindering successful vaccine creation [32]. The peak age-specific incidence for severe RSV disease is between 2 and 4 months, a period when infants have immature immune systems [33]. This complicates vaccine development as the immune response in infants is often biased towards a Th2 response, which is less effective in combating viral infections [32,34]. The presence of maternal antibodies can also interfere with the effectiveness of the vaccine in infants. These antibodies can neutralize the vaccine antigens before they elicit a strong immune response in the child [32]. Also, the short time frame between birth and the first exposure to RSV limits the opportunity for effective vaccination before the child is at risk of severe disease. These challenges are compounded by the need to balance safety and efficacy in a vulnerable population. These challenges faced by early RSV vaccine development and technological advancement gave RSV researchers substantial information to better understand the complexities of the immune responses induced by RSV and enable them to refine RSV vaccine designs.

## 3. Current FDA-Approved RSV Passive Immunization for Children

**Figure 1:**
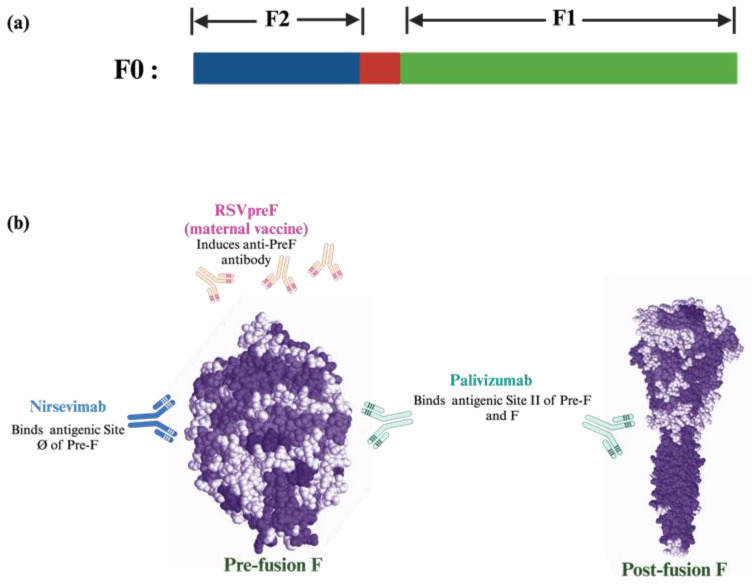
Prophylactic approach of RSV vaccine and mAb for children. (**a**) RSV F protein initial precursor (pre-F; F0), which is later cleaved by a furin-like protease into F1 and F2 that form the post-fusion conformation. The Pre-F protein structure was modeled using 5K6C of the RCSB Protein Data Bank [40] and post-F protein using 3RRR of the RCSB Protein Data Bank [41]. (**b**) Prophylactic mAb (palivizumab and nirsevimab), and the maternal vaccine (RSVpreF, Abrysvo) targeting different antigenic sites of RSV pre-F and post-F protein. (Created with Biorender.com).

Compared to the G-protein, the F protein has been more explored as a vaccine design target because its conservation among RSV subtypes is thought to be more potent in inducing neutralizing antibodies against RSV. The F protein has three different conformations: the inactive precursor form F0 (Pre-F), which forms a trimer and is then cleaved by furin-like host protease [35] to give the cleavage product F1 and F2 subunits. F1 and F2 subunits are covalently linked by a disulfide bond [36]. Several studies have reported that RSV-neutralizing antibodies (nAbs) that bind to the antigenic sites found only in the Pre-F conformation are more potent than the nAb that binds to the antigenic site found on both conformations [37,38]. Understanding the structure of the different conformations of the F protein and the development of the stabilized construct of Pre-F has led to the advancement and development of RSV vaccines and monoclonal antibodies (mAbs) that target both the pre-F and post-F conformation of the RSV F protein (Figure 1). Currently, the approved prophylactic treatments against RSV in children include two mAbs (palivizumab and nirsevimab) and a vaccine administered to pregnant women to provide passive immunization to newborns (Table 1). The description of other mAbs like motavizumab and motavizumab-YTE have been excluded, since the development of these mAbs has been discontinued and not approved by the FDA [39].

### 3.1. Palivizumab

Palivizumab (Synagis) is a humanized mAb (IgG1) developed through recombinant DNA technology that acts by binding to an epitope in the antigenic site II of RSV F protein [49], thereby blocking its fusion to the target cell and, in turn, blocking viral replication. Palivizumab was FDA-approved in 1998 and has been used for over two decades as a preventive drug against RSV for high-risk infants [50,51]. It is given to infants born prematurely (<36 weeks of gestation) and 6 months or younger during their first RSV season, infants diagnosed with bronchopulmonary dysplasia (BPD), and infants with congenital heart disease to prevent lower respiratory tract disease caused by RSV. In preterm infants, palivizumab has been reported to significantly reduce wheezing days during their first year of life, even after treatment [52], and decreased RSV hospitalization rates in high-risk preterm infants, with a 70% lower hospitalization rate compared to non-prophylaxed infants [53,54]. However, palivizumab offers only temporary protection; therefore, a single dose has to be given consecutively every month for the first five months of RSV season. This multiple dose administration poses challenges, including financial burdens and the stress of repeated hospital visits, which may also expose infants to other infections [55]. Moreover, the use of palivizumab is restricted to high-risk patients, leaving the needs of other pediatric patients and even the elderly unmet [56]. In the quest to overcome the limitations of palivizumab, a refined form named Narsyn was generated. It is a form of the antibody that is nasally administered to induce mucosal immunity in preterm infants. However, it failed its phase 2 clinical trial, as the daily intranasal administration to late preterm infants did not prevent RSV infection [57].

### 3.2. Nirsevimab

Nirsevimab is a humanized murine mAb approved in July 2023 for treating RSV lower respiratory tract infection (LRTI) in neonates and infants. Unlike palivizumab, nirsevimab has an extended half-life of 68.7 ± 10.9 days 36 [58,59,60]. The extended half-life of nirsevimab, defined by its modified Fc region [46,61], allows for just a single dose administration throughout an RSV season. Nirsevimab targets the pre-F protein of RSV, specifically the site Ø, which is highly conserved among RSV strains, which enhances its efficacy [45,62]. Several clinical trials led to the successful use of the mAb. In a clinical trial (NCT03979313), nirsevimab showed an efficacy of 74.5% for medically attended RSV-associated LRTI and 62.1% for hospitalization due to RSV-associated LRTI. This trial concluded that a single injection of nirsevimab before RSV season protected healthy late-preterm and term infants from medically attended RSV-associated LRTI 33. In another clinical trial (NCT05437510), nirsevimab showed an efficacy of 83.2% for RSV-associated LRTI, an efficacy of 75.7% for very severe RSV-associated LRTI and 89.6% efficacy against hospitalization for RSV-associated LRTI [63]. Nirvesimab was concluded to protect infants against hospitalization for RSV-associated LRTI and very severe RSV-associated LRTI. One dose of nirsevimab is recommended for all infants aged less than 8 months and born during or just entering their first RSV season and for infants and children aged 8–19 months who are at increased risk for severe RSV disease and entering their second RSV season.

### 3.3. RSVpreF Vaccine (For Pregnant Women)

RSVpreF vaccine is a bivalent protein subunit vaccine manufactured under the tradename Abrysvo ^®^ [64]. It was approved in May 2023 for the elderly and in August 2023 for pregnant women. The vaccine contains the RSV pre-F protein from the two major circulating antigenic subgroups RSV-A and RSV-B [65,66] but does not contain an adjuvant [66,67]. It is approved and recommended for pregnant women between 32 and 36 weeks of pregnancy [68]. Maternal immunization effectively protects the infant for the first 6 months of life. In the trial (Study ID: NCT04424316), where participants were pregnant women, the vaccine was well tolerated, and there were no safety concerns for the women or infants. The vaccine efficacy (VE) was 81.8% within 90 days after birth and was concluded to confer protection against severe RSV-associated LRTI in infants. At 180 days after birth, the vaccine had a reduced efficacy of 69.4% [69].

## 4. RSV Passive Immunization Under Development

**Figure 2:**
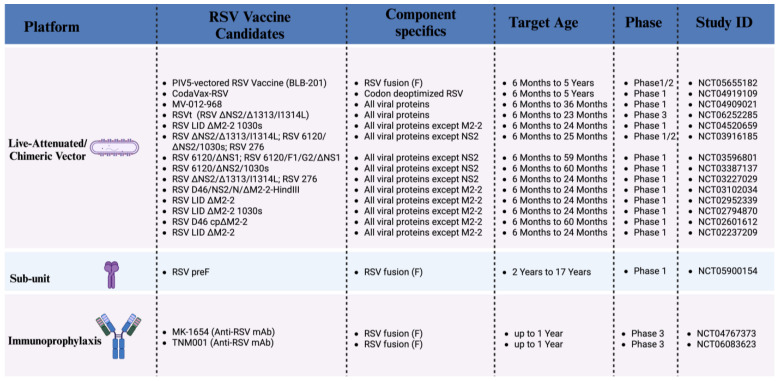
RSV vaccines and mAbs for children in clinical trials. Active clinical trials for pediatric RSV vaccine and mAb therapies with outlines of the platforms and components utilized in each therapy, the corresponding trial stages, target age groups, and clinical trial identification numbers. (Created with Biorender.com).

Currently, two additional prophylactic mAbs are in clinical phase 3 (Figure 2). The MK-1654 (clesrovimab) is a mAb developed as a single-dose intramuscular immunization for healthy pre- and full-term infants. This mAb targets site IV of the RSV F-protein and has an extended half-life. The results of the phase 1 clinical trial for MK-1654 in healthy adults reported its tolerability and a good safety profile similar to placebo [70], allowing the mAb to progress to phase 2b/3 (NCT04767373), which recently concluded in July 2024, and the results are expected very soon. One of the advantages of the MK-1654 mAb over those currently approved is its ability to penetrate the nasal epithelial lining fluid, which enhances its potential to provide more robust early protection against pediatric RSV infection by targeting the primary site of infection, which is the respiratory epithelium [71]. Additionally, this could offer infants better protection by promoting the induction of mucosal immunity giving children additional protection against RSV. Another mAb under study is TNM001 (trinomab), which targets the RSV pre-F protein. The phase 3 study (NCT06083623) is evaluating the efficacy, safety, and neutralizing antibodies in early- and mid-term preterm infants (<35-week of gestational age) and late preterm or full-term infants (≥35-week of gestational age) just entering their first RSV season.

## 5. RSV Pediatric Vaccines Currently in Development (Phase 1, 2 Clinical Trials)

Different therapeutic strategies have been studied to develop an effective RSV vaccine for children. Some of them represent promising opportunities to prevent the disease. The most recent approaches described are live attenuated vaccines, recombinant vector-based vaccines, protein-based vaccines, nucleic acid-based vaccines, and monoclonal antibodies (Figure 2).

### 5.1. Live Attenuated Vaccines

The adverse results of the trials with the formalin-inactivated RSV vaccine demonstrated poor protection and enhanced RSV disease, showing the complexity of the immune response and the importance of the course of the disease. Since then, live attenuated vaccines have been introduced as a promising strategy. They use a weakened form of the virus that is still alive but has been modified so that it cannot cause severe disease in healthy individuals. In principle, these vaccines closely mimic a natural infection, providing robust and long-lasting immunity. Earlier attempts to develop a live attenuated RSV vaccine identified cold-passaged (cp) and temperature-sensitive (ts) mutations and deletions (Δ) of nonessential genes that, when combined, resulted in attenuated vaccine candidates. With the development of reverse genetics, generating complementary DNA-derived RSV vaccine candidates with predetermined combinations of attenuating mutations became possible. This method has been used to incrementally adjust the attenuation level of RSV to produce candidates with the potential to be well tolerated and immunogenic in young infants. Subsequently, several live attenuated vaccines have been developed by deleting some of the viral proteins. Most live attenuated vaccines are administered intranasally, such as the G protein-deleted vaccine [72], the M2-2 protein-deleted vaccine [73], and the SH protein-deleted vaccine. Further, recent advancements in RSV live attenuated vaccine design have targeted some nonessential RSV proteins like the non-structural proteins NS1 and NS2. One of the important advantages of the intranasally administered live attenuated vaccine over other approaches is that it is given at the replication site, which helps to induce a broad immune response. In general, live attenuated RSV vaccine development attempts have shown promising results, with some vaccines inducing strong immune responses and showing potential efficacy in RSV-naive children [74]. However, none of these vaccines have received regulatory approval to date.

One of the candidates of live attenuated designs is the intranasal vaccine named RSV/ΔNS2/Δ1313/I1314L, which is generated by deletion of the interferon antagonist NS2 gene and introduction into the L polymerase protein gene of a codon deletion (Δ1313) that confers temperature-sensitivity and is stabilized by a missense mutation (I1314L). Additionally, four amino acid changes in the F protein increase the stability of infectivity by favoring the immunogenic pre-fusion conformation. The result of the clinical trial NCT01893554 showed the vaccine to be a genetically stable candidate as an RSV vaccine and immunogenic in RSV-seronegative children, which makes it an active candidate in development [75,76,77]. Using a similar strategy, a cDNA-derived version of RSV subgroup A, strain A2 vaccine named LIDΔM2-2, is a version with 241 nucleotides deleted from the M2-2 ORF and several deletions on the SH gene [78]. M2-2 is an RSV protein that mediates a switch from transcription to RNA replication, which enhances mRNA synthesis early in infection. M2–2 deletion in vaccine development has helped attenuate viral growth [79], as it plays a crucial role in virus assembly, budding, and the formation of virus particles [80]. The clinical trial NCT02237209 evaluated the safety and immune response to the vaccine in RSV-seronegative infants and children. However, a stabilized temperature sensitivity mutation 1030s tested in the clinical trial NCT02794870 resulted in better durable immunity in seronegative children [81]. Another active live attenuated vaccine candidate in the phase 1 clinical trial (NCT03387137) is RSV/6120/ΔNS2/1030s. This vaccine candidate is a cDNA-derived live vaccine attenuated by two deletion mutations. The first protein deletion, NS2, is the well-known interferon antagonist gene, and the second deletion is the codon 1030S in the polymerase gene, conferring temperature sensitivity [22]. A single intranasal dose has been demonstrated to be immunogenic and genetically stable. However, the presence of rhinorrhea was associated with this vaccine. The vaccine candidate named MV-012-968 (NCT04909021) is also under development, and it is administered intranasally. It is a highly attenuated vaccine in which the codons of the NS2, NS1, and G genes have been optimized, and deletion of the SH gene has been demonstrated to have protective effects [82]. Additional live attenuated vaccine candidates in early development are listed in Figure 2.

### 5.2. Recombinant Vector-Based Vaccines

A recombinant vector-based vaccine delivers the gene of relevant RSV proteins inserted in other viruses’ backbones to induce a humoral and cellular immune response. These vaccines mimic natural RSV infection, helping the immune response recognize and respond to the actual pathogen if the patient is exposed to it in the future. An example of a vector-based vaccine is Ad26.RSV.preF, a recombinant, replication-incompetent adenovirus type 26 (Ad26)-based RSV vaccine encoding a conformation-stabilized RSV pre-fusion (pre-F) protein [83]. This vaccine has already been tested in adults and has shown an efficacy of 80% in preventing more severe illness by RSV infection [84,85,86,87,88,89]. However, the development of this vaccine (trial NCT03982199) has been terminated early due to a strategic reprioritization by the sponsor (Jansen Vaccines & Prevention B.V.), according to ClinicalTrials.gov. Aside from the terminated Ad26.RSV.preF vaccine, an active trial is underway for the PIV5-vectored RSV Vaccine (BLB-201). This vaccine candidate is a live, virally vectored RSV vaccine based on parainfluenza virus 5 (PIV5) encoding the RSV F antigen. Preclinical studies of the PIV5-vectored vaccine for RSV have shown that the vaccine induces an RSV-F-specific antibody and cell-mediated immunity in mice and African green monkeys and showed protection when challenged with RSV [90]. The promising safety and immunogenic ability of the vaccine has advanced it to a clinical phase 2 trial (NCT05655182) being carried out in infants (8 to 24 months of age) and children (18 to 59 months of age).

### 5.3. Protein-Based Vaccines

The protein sub-unit vaccines use harmless pieces of the target pathogen (often viral proteins) to stimulate an immune response. These vaccines introduce these proteins directly into the body rather than using a live or inactivated virus. The immune system recognizes these proteins as foreign, generates antibodies against them, and develops memory on how to combat them in the future. Two recent FDA-approved vaccines, the RSV pre-F for pregnant women and GSK3844766A (RSV PreF3 OA) for the elderly, are recombinant subunit vaccines [91,92]. These two vaccines have demonstrated efficacy in preventing the disease, while the maternal vaccine provides passive immunization for the newborn [93]. Currently, the only protein sub-unit vaccine for children is RSV pre-F and is in phase 1 clinical trial (NCT05900154). The trial is being carried out in children aged 2 to <18 years.

### 5.4. Nucleic Acid-Based Vaccines

Nucleic acid-based vaccines, such as mRNA vaccines, represent a modern approach to immunization. These vaccines work by delivering genetic material that encodes for a specific antigen of a pathogen, allowing the body to produce the antigen itself, which then triggers an immune response. There were previously two mRNA vaccines (mRNA-1345 and mRNA-1365) for children in a clinical trial. mRNA-1345 is an RSV-specific vaccine, while mRNA-1365 is an RSV/human metapneumovirus (HMPV) combination vaccine. The vaccine was against RSV F protein and was delivered as a lipid nanoparticle-encapsulated mRNA. A phase 1 trial study report showed that the mRNA was well tolerated at all doses tested. After one month post-vaccination, the mRNA vaccine was shown to boost the RSV-specific neutralizing antibody and RSV prefusion binding [25]. The antibody level, when measured after 6 months post-vaccination, was above the baseline. Based on the positive results of the phase 1 study, the vaccine advanced to clinical trial phase 2 (NCT06097299) in children ages 5 to <24 months. However, some safety issues were recently discovered in two vaccinated infants who tested positive for RSV and contracted a severe lower respiratory tract infection (LRTI). As a result, the vaccine trials were halted to prevent a potential RSV vaccine-associated enhanced respiratory disease (VAERD) as was previously encountered in the 1960s.

## 6. Advanced-Stage RSV Pediatric Vaccine Candidate (Phase 3 Clinical Trial)

There are several promising RSV vaccines for children as they are currently in their late phase (Phase 3) of clinical trials (Figure 2). One of the lead candidates is the Respiratory Syncytial Virus Toddler (RSVt) live-attenuated (RSV/ΔNS2/Δ1313/I1314L) vaccine for children between 6 and 23 months old. This vaccine candidate has a deleted non-structural protein 2 (NS2), which could contribute to its safety. NS2 is an RSV-encoded protein that interferes with and downregulates type 1 and type 3 interferon induction [94,95]. NS2 also has a pathogenic effect of causing epithelial cell shedding and airway obstruction in both in vitro and animal studies [96]. The codon 1313 in the polymerase (L) gene was also deleted, which gives the vaccine its mild temperature sensitivity, and leucine (L) at codon 1314 was substituted for isoleucine (I) to help stabilize the deletions genetically and phenotypically [97]. The attenuation phenotype of this candidate vaccine was tested in chimpanzees, and the data showed that the virus was highly attenuated and had a low-level replication [97]. The safety and immunogenicity of this vaccine candidate were tested in a phase 1 clinical trial (NCT01893554) [75]. Estimated data on vaccine efficacy collected from the vaccinated children showed that the vaccine candidate had an efficacy of 67% against RSV-associated medically attended acute respiratory illness (RSV-MAARI) and 88% efficacy against RSV-associated medically attended acute lower respiratory illness (RSV-MAALRI) [98]. This vaccine candidate has advanced to clinical phase 3 (study ID: NCT06252285). The study is being conducted in an estimated 6300 children aged 6 months to 22 months. The study evaluates the immunogenicity, efficacy, and safety of RSVt vaccine. The study plan for the phase 3 trial, which was first submitted in January 2024, is expected to last 24 months for each participant.

## 7. Conclusions and Future Direction

Developing an effective pediatric RSV vaccine remains a significant challenge, necessitating a focused effort to overcome obstacles specific to this population. Several clinical trials at different phases are currently ongoing for pediatric RSV vaccines. Still, one of the challenges is that many of these trials are only conducted in children aged 6 months and older, whereas the age at which children are most susceptible to RSV lower respiratory tract infection is under 6 months of age. More studies need to be conducted on other strategies for combating RSV infection in children. One of the emerging fields of immunotherapy is personalized immunotherapy, which focuses on tailoring treatments to enhance immune responses in vulnerable populations like immunocompromised individuals. Current studies focus on developing targeted T cell therapy using RSV-specific T cells from healthy donors, aiming for personalized immunotherapy in high-risk populations like immunocompromised individuals such as hematopoietic stem cell transplant (HCT) recipients [99]. Personalized immunotherapy against RSV for children could also be explored for potential breakthroughs, although high costs remain a significant limitation. New mAbs, such as nirsevimab, have shown extended protection against RSV. Further advancements could focus on improving monoclonal antibodies against RSV with even more extended protection than nirsevimab, to ensure protection for those children who have aged beyond the timeframe for receiving nirsevimab. Overall, the progress toward developing an efficient RSV pediatric vaccine is highly promising, with a potential RSV vaccine for infants and young children available by 2026.

## Figures and Tables

**Table 1:** Currently FDA-approved passive immunization for children.

Immunization	Target Population	Mean Half Life	Duration of Protection	Target Protein	Ref.
Palivizumab	High risk and Preterm infants (born <36 weeks of gestation)	~20 days	~30 days	Antigenic site II of the F protein	[42,43,44]
Nirsevimab	8-month-old infants (born during RSV season or entering their first RSV season).	68.7 ± 10.9 days	~150 days	Site Ø on the RSV pre-F protein and neonatal Fc receptor (FcRn)	[45,46,47]
RSV PreF vaccine	Pregnant women between 32 and 36 weeks of pregnancy		Up to 6 months after birth	RSV prefusion F protein	[48]

## Data Availability

The reference sources supporting the conclusions of this review manuscript are all present within the article.
